# Application of 0-1 test for chaos on forward converter to study the nonlinear dynamics

**DOI:** 10.1038/s41598-022-19667-7

**Published:** 2022-09-20

**Authors:** Ahsan Ali, Sajid Iqbal, Hafiz Abdul Muqeet, Hafiz Mudassir Munir, Syed Sabir Hussain Bukhari, Jong-Suk Ro, Zeeshan Akbar

**Affiliations:** 1 Department of Electrical Engineering, University of Engineering and Technology (UET), Lahore, 54890 Pakistan; 2 Department of Mechatronics and Control Engineering, University of Engineering and Technology (UET), Lahore, 54890 Pakistan; 3 Department of Electrical Engineering Technology, Punjab Tianjin University of Technology, Lahore, Punjab 54770 Pakistan; 4 Department of Electrical Engineering, Sukkur IBA University, Sukkur, Sindh Pakistan; 5 School of Electrical and Electronics Engineering, Chung-Ang University, Seoul, South Korea

**Keywords:** Electrical and electronic engineering, Energy infrastructure, Energy science and technology

## Abstract

DC–DC converters has significant role in the applied power electronic systems, distributed power systems, computers, home appliances and communication equipment. A converter must remain within the specified range of operation. The main goal of this paper is to discuss the nonlinear behavior of forward converter and highlighted the application of the 0-1 test by applying it on the forward converter. As forward converter may contains electronic components, which cause instability in the system. So, it is necessary to understand its behavior when specifications of components are changed. To study chaotic behavior, 0-1 test will be applied on the forward converter, which is a novel technique outperform in unearthing the subtle chaotic behavior in deterministic dynamical systems. The forward converter goes from period-1, period-2, period-4 and finally become chaotic when the load resistance is varied. This variation in the behavior of the forward converter are analysis through 0-1 test for chaos. Moreover, time series plot, phase portrait and Bifurcation diagram for forward converter is also drawn for the validation of results obtained from 0-1 test. Test algorithm is applied via MATLAB and simulation of forward converter via MultiSim by varying its load resistance.

## Introduction

The complex behavior of the system sometime describes by the word chaos. Chaos is one type of characteristics shown by the by complex systems. Quasi-periodicity and subharmonics are other kinds. Nonlinear dynamics are generally deal with the field of science in which dynamical behavior of the nonlinear system is discussed^1^. A differential equation usually defines the nonlinear system. Nonlinear system have a pivotal role in the analyses of natural phenomenon but in last few decade it also gains importance in the engineering research field^2^. Nonlinearities in the power electronics is the key challenge to the engineer in modern era. The understanding of the chaos is necessary for every power electronics engineer. During the early stage of power electronics development nonlinear phenomenon quasi-periodicity, chaos and harmonics appeared while experimentation. The regular periodic operation is the primary objective of the power electronics engineer, so to avoid any unpredictable strange operation, the circuit parameters that creating problem or chaotic behavior need to be adjusted. Such adjustment of circuit parameter is done by trial and error method^3^. However, we require better understanding of circuit operation at various point to get more reliable design. Moreover, it may acquire new possible operating regimes of power electronic system after getting enough understanding of circuit. Power converters exhibit chaotic behavior. Chaos is an unpredictable long-term behavioral disorder showed by the nonlinear dynamical system. In order to get knowledge about this phenomenon, various tools have been established^4,5^ Bifurcation diagrams are the well-known evaluation tool for the analysis of nonlinear system when one or more parameters are 7 changed. It is usually plotted in 2D plane by placing variable on x–y axis and varying variable on x-axis^6^. To draw higher order bifurcation diagram, extensive computation is involved. Power electronic use the discrete model to generate the bifurcation diagram through state variable^7^.

**Figure 1:**
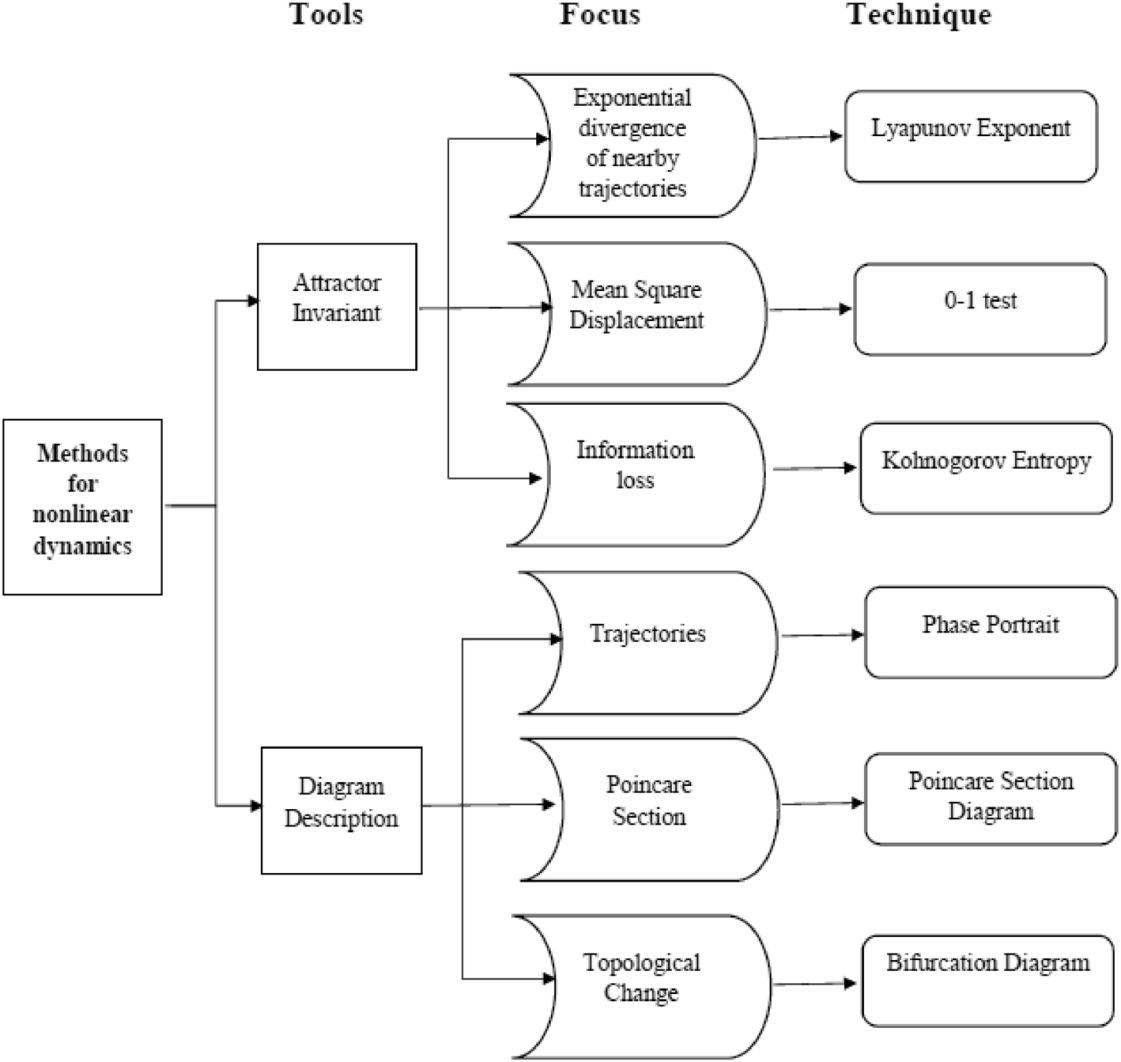
Nonlinear dynamical methods for times series data^8^.

The power electronics converters test by various techniques are summarized as below in Fig. 1. A phase portrait shows convergences and divergence trajectory towards the stable point^7^. Time domain waveforms show the steady state and the transient response.The time series complexity of power converters has been observed through various techniques. These methods were discussed in literature. These techniques are divided into three main groups, i.e. methods derived from nonlinear dynamics entropy and fractality^8^. The method used in this research to determine the nonlinear dynamics of forward converter are 0-1 test, bifurcation diagram, Poincare map, and phase portrait is summarized shown in the Fig. 1.

The chaotic and regular behavior of the deterministic dynamical system are studied through 0-1 test for chaos. Unlike other test this test is simple, and it does not need to know the nature of the system. This test is easily applicable on the partial differential equation, ordinary differential equation, or experimental data. The test is binary in nature it gives result 0 or 1, 1 for the chaotic system and 0 for the periodic system^9,10^. This tool is widely used for detecting chaos in diverse field due to the ease in its implementation, wide range of application and evaluation. Few examples where 0-1 testing is used recently on experimental data to discrete or continuous time system^11^.

To detect chaos in a real-world system different technique are used. Lyapunov exponent is the one of them which is extremely sensitive to the noise^12^. Therefore, in many cases it is very difficult to implement. Lyapunov exponent requires phase space reconstruction to distinguish between the chaotic and regular dynamical system^13^. After that many other different algorithms have developed and the most prominent one is 0-1, which directly applied on the time series 0-1 test developed by Gottwald and Melbourne which is not affected by noise^10^. This method takes time series data as input and gives 0 or 1 as the output according to the system dynamics: 1 for chaotic and 0 for regular. Moreover, Phase space reconstruction does not require in case of 0-1 test. This paper focuses on the nonlinear behavior of the forward converter, which is DC-DC converter. The variations and complications of nonlinear circuit and every converter topology show different problem which need to be examined. In this research work, forward converter is studied thoroughly through different nonlinear techniques like 0-1 test, bifurcation diagram, Poincare map, time series plot and phase portrait plot. The complex chaotic behavior of the system will be explored by varying control parameter. The simulation results will obtain from circuit simulator software. The experimentation and simulation result will then carefully observe to understand the nonlinear behavior of the system.

## Literature review

The nonlinear dynamics of the power converters are major concern for the engineers and researchers. The scholars studied chaos phenomenon in the power converters to understand their behavior. The research help to enhance the efficiency of the power converters and it also benefit for industries.

The basic converter topologies i.e. Buck, Boost and Buck-Boost converter have significant importance in converter technology. The research work had done on nonlinearity in buck, boost, and buck-boost converter topology. In this thesis^7^ pulse width modulation (PWM) buck and boost switching regulators simulated and carried out the nonlinear analysis. The buck-boost converter operated under the peak current control mode, the storage energy in this dc system, used to examine the phenomena of nonlinear, chaos and bifurcation observed in^14^. This paper^15^ took current and voltage controlled buck converter for investigation of nonlinear phenomena and observed the bifurcation and chaos. The buck converter fed with the rectifier having ripple were studied in^16^ and discussed chaos phenomenon and bifurcation. In^17^ the author studied the chaos phenomenon in three topological power converters having closed loop. In^18^ the author showed the coexistence situation in flyback converter: two period orbits , period-1 and period-2 orbits and chaotic and period-1 orbits and lastly chaotic and period-2 orbits.

Flyback converter is one the most important in converter in power converters used in daily life. The relationship between iterative nonlinear mapping and dynamical system like DC-DC converter is discussed by David et al. in^19^ Flyback converter also produce nonlinearity at some point, the phenomenon of chaos in a UC3842 current programmed flyback converter was discussed in^20^. In this operation principle, mathematical models and discrete-time analytic solution derivations of the flyback converter were presented. Fei-Hu et al.^21^ explored the nonlinear behaviors in the UC3842 current-mode controlled flyback converter when the switching frequency altered. Ru Yang et al. had established the duplicate symbolic sequence of voltage-mode-controlled flyback converter and it had been applied as an example to identify various types of the bifurcation, in the bifurcation process of the system^22^. The duffing oscillator is the well known nonlinear circuit, the studied about critical situation of the generating of the chaos by fractional derivatives and nonlinear damping in the duffing oscillator was examined by Wang et al.^23^. The system stability affecting by the fraction order term and non-linear factor. This influence on the system stability is studied using piecewise nonlinear oscillator^24^.

The nonlinearity was observed in forward converter by various researches. Bi et al. studied nonlinearities in forward converter by changing the values of load resistance, filter inductor and filter capacitor^25^. Discrete iterative mapping of voltage-mode controlled forward converter developed to explore the nonlinearity and obtained the bifurcation diagram with the input voltage, reference voltage and load resistor as bifurcation parameters^26^. Jingmei et al. done reduction of EMI through chaos, for that purpose voltage-mode controlled forward converter circuit is used by Bi et al.^27^. Wei et al. discussed nonlinearity in forward convert through bifurcation phenomena when voltage regulated forward converter operated on both continuous and discontinuous mode^28^. Half-bridge converter was analyzed by Song et al.^29^ and its dynamics were studied by the author.

The nonlinear dynamics of system study through various technique like bifurcation diagram, Kolmogorov entropy, minimum embedding, correlation dimension, Poincare Map, Lyapunov exponent and many others. 0-1 test is one of the such method.The 0-1 test method is new technique to determine the chaotic and regular dynamics does not require phase space reconstruction^30^. This method takes time series data as input and gives 0 or 1 as the output according to the system dynamics whether it is chaotic or regular^10,31,32^.The two well-known system Rössler and Lorenz and two less-known system calcium oscillatory models and arc circuit are used to utilize in the comparison of the determination of the periodic and chaotic oscillation by sample entropy and 0-1 test for chaos^33^. The combination of the approximate entropy and 0-1 test for chaos is introduced in order examined the nonlinearity of the three degree of freedom mechanical system^34^. Adel et al. studied the chaotic behavior of the Fractional-order Arnold map by 0-1 test for chaos^35^.

Moreover, at the beginning the 0-1 test was implemented on the logistic map driven^10^, damped Kortweg de Vries equation and forced van der Pol^36^. Kim implement the 0-1 test on Local K spectrum of non-chaotic strange attractor^37^. Karsten Webel applied 0-1 test on the data obtained from German stock exchanges to verify results^38^. Falconer et al. analyzed the chaotic behavior of bipolar motor and studied the effectiveness of 0-1 test on experimental data^39^. Zachilas et al. briefly discussed about the implementation of 0-1 test on Hamiltonian systems they presented four Hamiltonian system two even and two odd order^40^ and their results were matched with 0-1 test. Baogui Xin et al. presented a discrete complex interaction model about industrial production and environmental quality in a closed area and then applied 0-1 test in order to validate the chaotic phenomenon of their model^41^. A simple method of detection chaos tool was presented by Daniel et al. by combining the several such tool of detecting chaos mainly 0-1 test and presented it implementation heart rate variability^42^. Michelle et al. applied the 0-1 test for chaos on an Aeroelastic System and compared the results with the attractor reconstruction method such as Lyapunov exponents^43^.

Furthermore, the 0-1 test gained lot of more attention because of its simplicity and research used it to study the chaotic dynamics of various systems. Adel Ouannas et al. used 0-1 test, entropy, bifurcation diagram and Co-complexity to analyzed the chaos in discrete fractional duffing system^44^. In this article the author discussed about the false negative result of the 0-1 test and presented ideas about how to avoid such result^45^ . In this paper^46^, two new techniques were discussed which are center of gravity and box counting. They also thoroughly studied about benefits and disadvantages of these methods. The dynamical behavior of the Henon–Lozi type map were studied by applying numerical tools which are: largest Lyapunov exponent, bifurcation diagram, 0-1 test and phase portrait. It displayed that the map shows a range of numerous dynamical properties from coexisting attractors to chaos^47^. This research work in^48^ explored the post-flutter nonlinearities because of the wing high aspect ratio. The results of aeroelastic system were then used to analyzed for the comparison of attractor reconstruction and 0-1 test for chaos.

## Methodology

### Overview of the binary 0-1 test

The 0-1 test is initially presented by Gottwald and Melbourne^10^. It is a binary test for chaos detection in dynamical system which does not require phase space reconstruction. It uses the time series of underlying system. The test has been successfully implemented to flows and maps, but in this study, it is implemented on the continuous system such as forward converter. The chaotic and regular behavior of the deterministic dynamical system are studied through 0-1 test for chaos. Unlike other test this test is simple, and it does not need to know the nature of the system.

**Figure 2:**
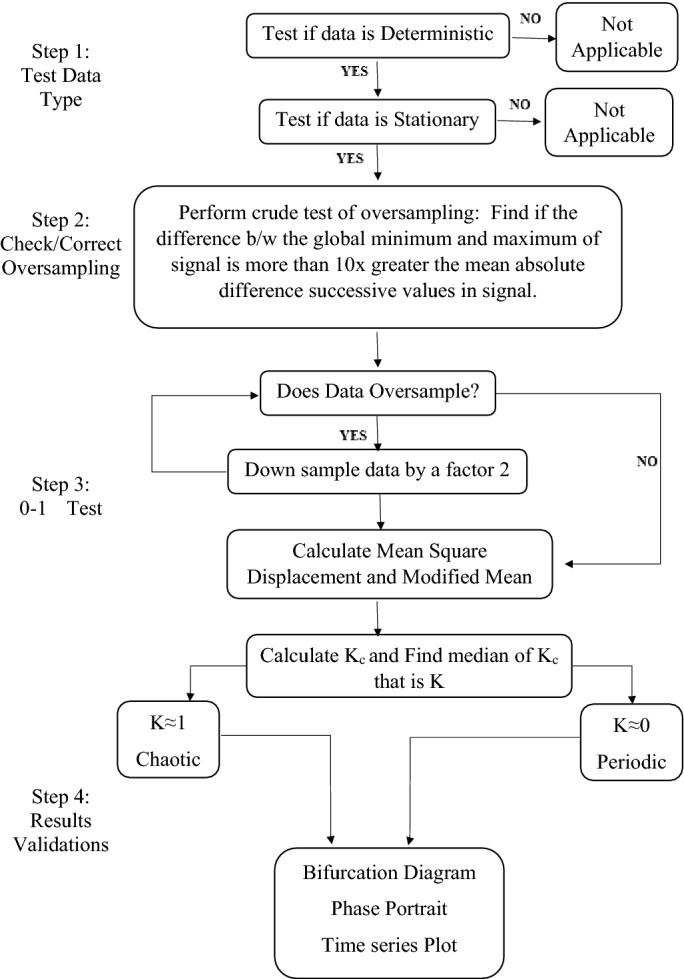
Flow chart of research methodology.

This test is easily applicable on the partial differential equation, ordinary differential equation, or experimental data. The test is binary in nature it gives result 0 or 1, 1 for the chaotic system and 0 for the periodic system^9,10,39^. This tool is widely used for detecting chaos in diverse field due to the ease in its implementation, wide range of application and evaluation. The flow chart how the research is conducted given in Fig. 2.

### Condition for the implementation of 0-1 test

For the implementation of 0-1 test the following conditions must be fulfilled to get the require result from the test.

1. Enough large time series data is required to adequately determine the asymptotic behavior of MSD^10,39^.
2. The short transient response of the system require to removes in order to avoid the false result and time series taken for test after removal of transient response^36,49^.
3. The data should be deterministic and stationary.

The 0-1 test may fails to produce result in some cases for example when the time series used to implement the test are obtained from the near edge of the chaos^32^. Moreover, when the dimension of the attractor is too large and extremely long transient the 0-1 test is impracticable^36^.

### Effects of number of data points

The number of data points have impact on the results of 0-1 test. To avoid the false results the number of data points must be addressed while implementing the results. Finite size effect in three ways^10^. The time series need to be large enough to explore the dynamics of the system this problem affecting all test of chaos. The limit $n\ll N$ while calculating the mean square displacement. So, we require to choose $\mathrm {n}_\mathrm {cut}\ll N$and $\mathrm {n}_\mathrm {cut}$ = N/10 The asymptotic behavior of $M_c (n)$ or $D_c (n)$ required $\mathrm {n}_\mathrm {cut}$ and N, which may require sufficiently large data. In case of small number of data points the asymptotic growth is not dominating to visualize the results.

### Effect of oversampling on continuous times series data

For continuous time series there is a well-known oversampling issue that must be addressed^10,31,50^.

1. One method which suggested by Gottwald and Melbourne is to observe the Poincare’s Section
2. A second, perhaps more usual, approach by visual inspection^30^.
3. Third method is more refined method discussed in^10,51^ use of the first minimum of mutual information.

## Simulation circuit of forward converter

**Figure 3:**
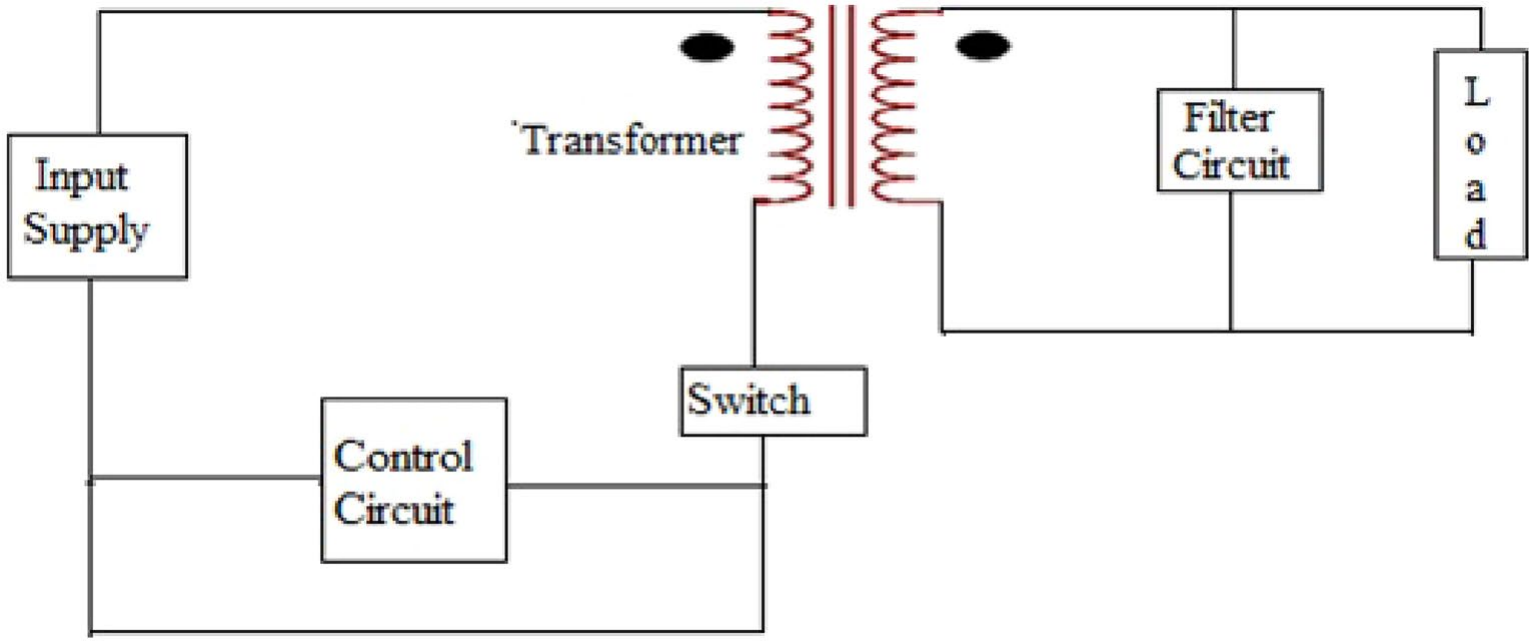
Schematic diagram of a current forward converter.

**Figure 4:**
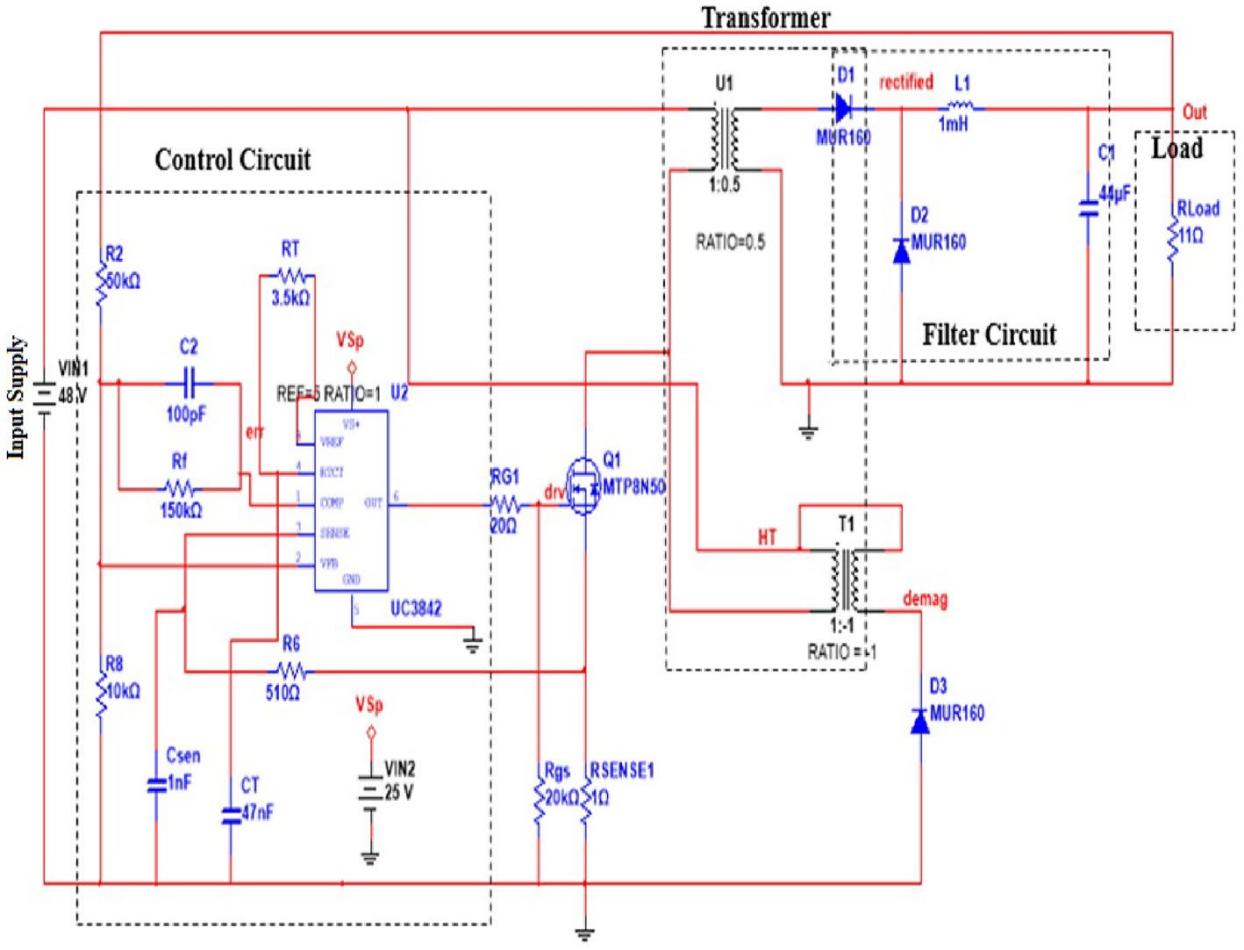
MultiSim simulation circuit of forward converter.

A forward converter has less output DC voltage than the applied DC voltage and its input and output is isolated from input by transformer. Figure 3 shows the schematic diagram of a current mode forward converter. Circuit in Fig. 4 shows the simulation circuit of forward converter. The circuit is simulated in the MultiSim. The converter is simulated first, then the waveform of the inductor current and output voltage is obtained. The phase portrait and bifurcation diagram of the converter are also drawn. The circuit parameters are given in Table 1.

**Table 1:** Circuit parameters of forward converter.

*Parameter*	*Values*
Input voltage ($\mathrm {V}_\mathrm {in}$)	48 V
Ouput voltage ($\mathrm {V}_\mathrm {out}$)	12 V
Switching frequency (F)	10 kHz
Resistance (R)	4–12 ${\Omega }$
Inductance (L)	1 mH
Capacitance	44 $\upmu$F
Turn ratio (Pri:Sec:Ter)	1 : 0.5 : 1

## Results and discussion

The simulation and experimentation circuits were shown in “Simulation circuit of forward converter”. In this section, results obtained through simulation and experimentation were analyzed through nonlinear techniques, which were 0-1 test, phase portrait, time series plot and bifurcation diagram discussed in “Methodology”.

### Verification of the 0-1 test via bifurcation diagram

**Figure 5:**
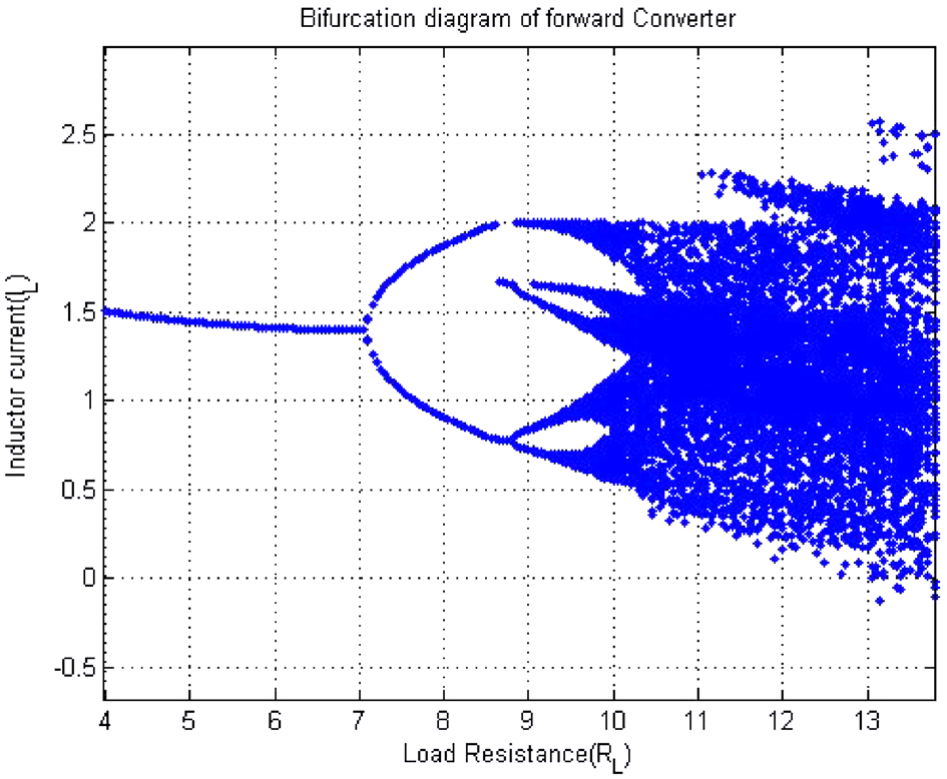
Bifurcation diagram of forward converter when $$ R_L$$ changing from $$R_L={4}\,{\Omega }$$ to $$R_L={12}\,{\Omega }$$.

**Figure 6:**
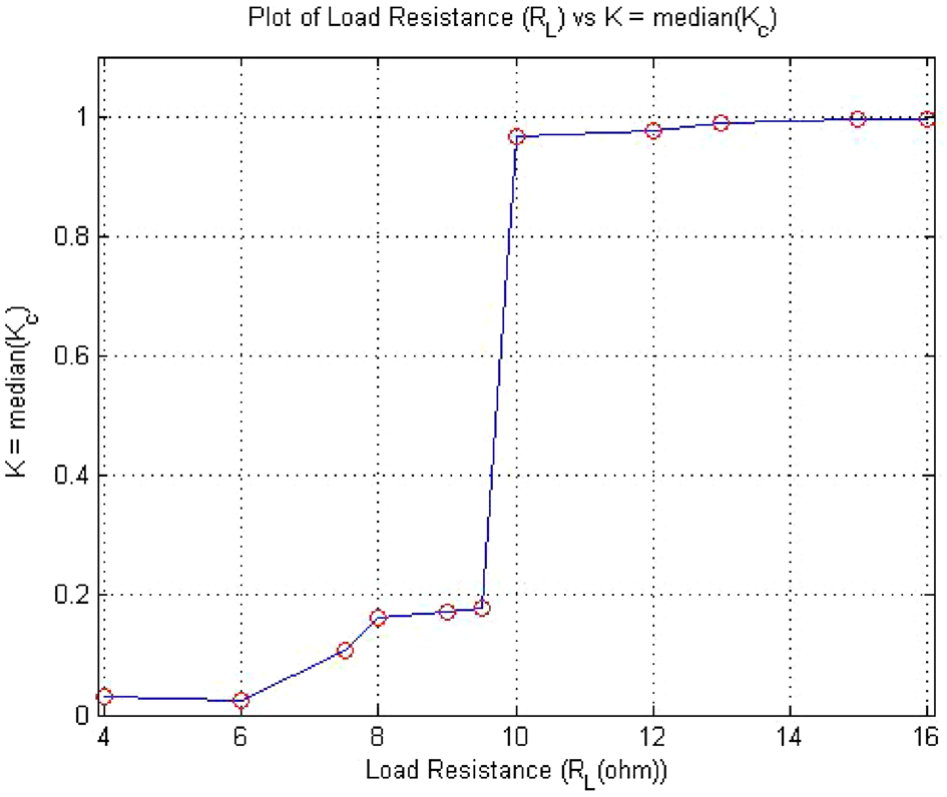
$$R_L$$ vs K plot of 0-1 test for forward converter when $$ R_L$$ changing from $$R_L={4}\,{\Omega }$$ to $$R_L={16}\,{\Omega }$$.

Period doubling route to chaos is a salient feature of chaotic systems. The dynamic chaotic system was observed through bifurcation phenomenon. How does chaos occur after a period? This phenomenon pictorially had observed through bifurcation diagram as shown in Fig. 5. The result obtained from test were clearly matched with bifurcation diagram shown in Figs. 5 and 6, which was validated 0-1 test. In Fig. 5, the value of k changing from 0 to 1 as the value of $R_L$ changing from $R_L={4}\,{\Omega }$ to $R_L={16}\,{\Omega }$. Similarly, with $R_L$ as the bifurcation parameter, the forward converter bifurcation diagram was drawn in MatLab. The current via inductor were considered for analyzing the bifurcation. $R_L$ was on x-axis and Inductor current $I_L$ on y-axis. Figure 5 shows bifurcation diagram of forward converter. The figure had clearly showed that period-1 at $R_L={4}\,{\Omega }$ , period-2 started at $R_L={7.5}\,{\Omega }$ , period -4 at $R_L={9}\,{\Omega }$ and chaos at $R_L={11}\,{\Omega }$ .

### 0-1 test results of forward converter

In this section, the result of the forward converter after the implementation of the 0-1 test were examined and discussed in detail.

#### 0-1 test results for forward converter at period-1

**Figure 7:**
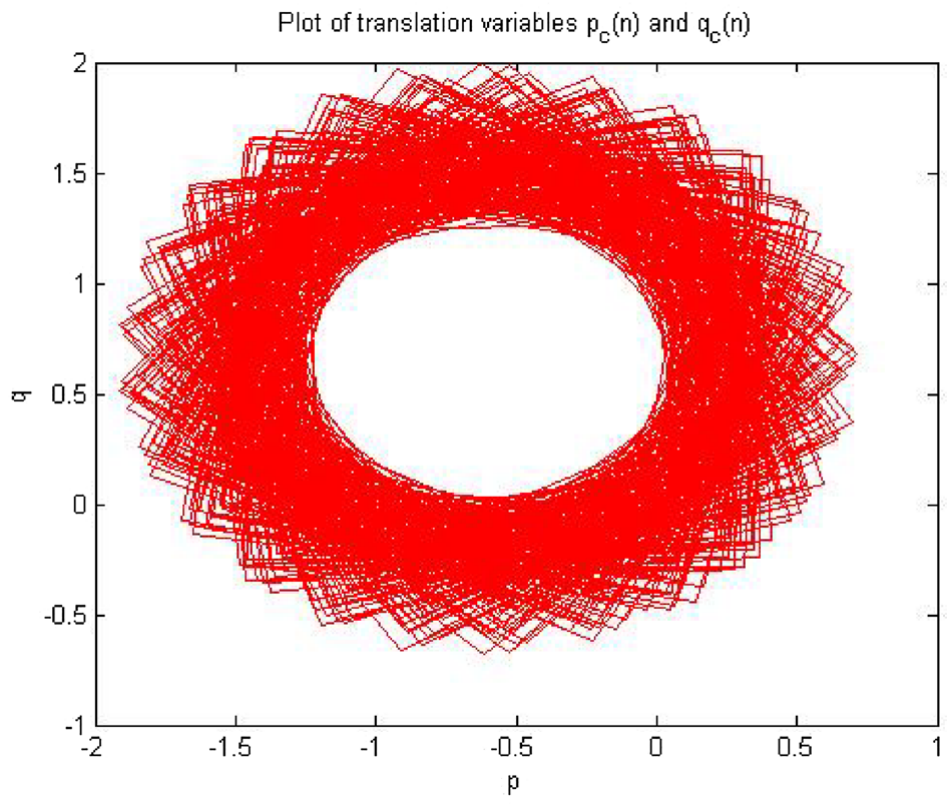
Plot p vs q showed bounded shaped hence its is periodic.

**Figure 8:**
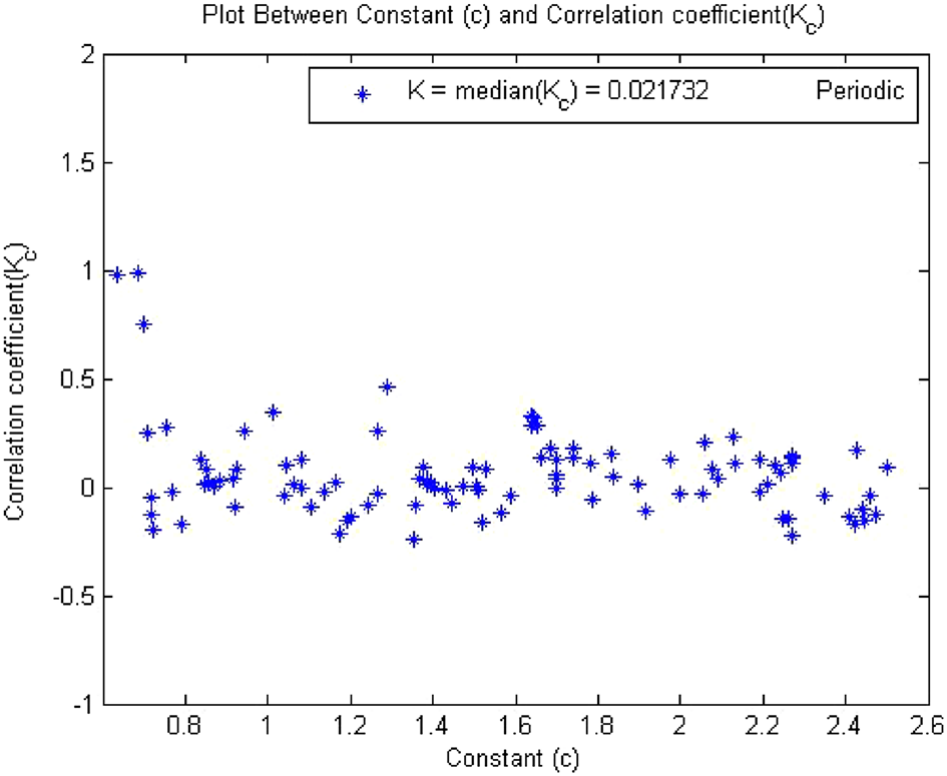
Plot of c vs $K_c$ shows periodic behavior as value of K = 0.02173.

**Figure 9:**
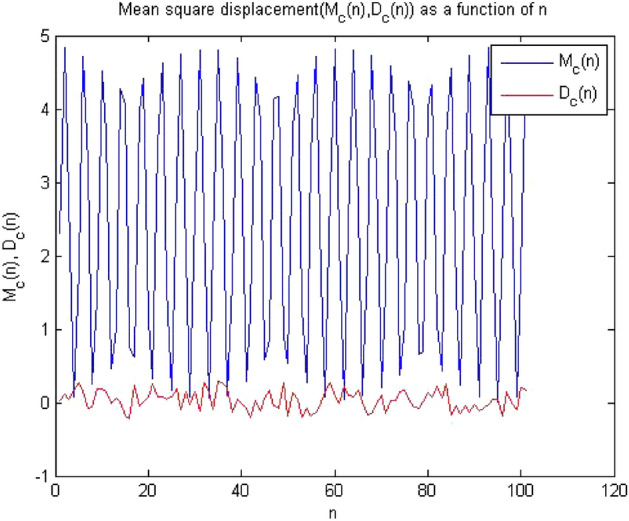
Plot of MSD vs time corresponding to periodic dynamics.

Times series data was acquired from the simulation circuit of forward converter at $R_L={4}\,{\Omega }$,which gave period 1. When 0-1 test algorithm was applied on this time series data which was discussed in “Methodology”, it gave the values indicator K = 0. The plot between p and q was bounded shown in Fig. 7. However, for periodic system the value of K must be near to zero and the value of K = 0.02173 for this time series shown in Fig. 8 and the mean square displacement plot did not show any asymptotic growth shown in Fig. 9. Hence, with the help of above results, the 0-1 test confirms that the time series was periodic. Therefore, forward converter showed periodic behavior when its load resistor was equal to ${4}\,{\Omega }$.

Figures 7, 8 and 9 showed the forward converter had periodicity in their behavior and all three plot support the argument made by the 0-1 test methodlogy.

#### 0-1 test results for forward converter at period-2

**Figure 10:**
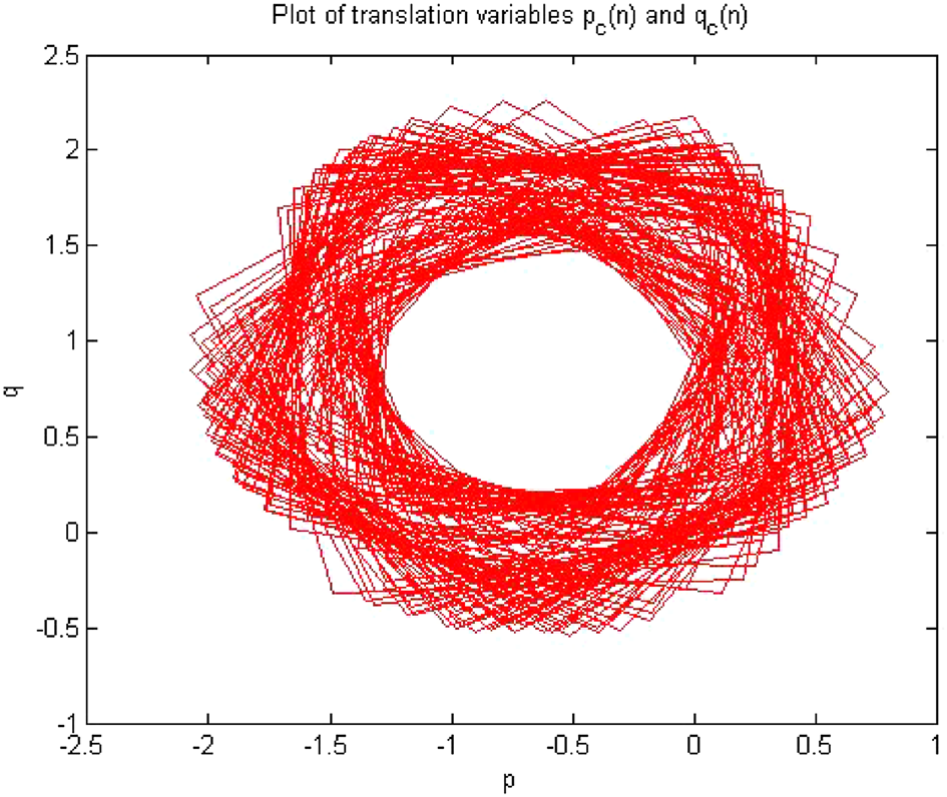
Plot of p vs q shows bounded shape having regular behavior.

**Figure 11:**
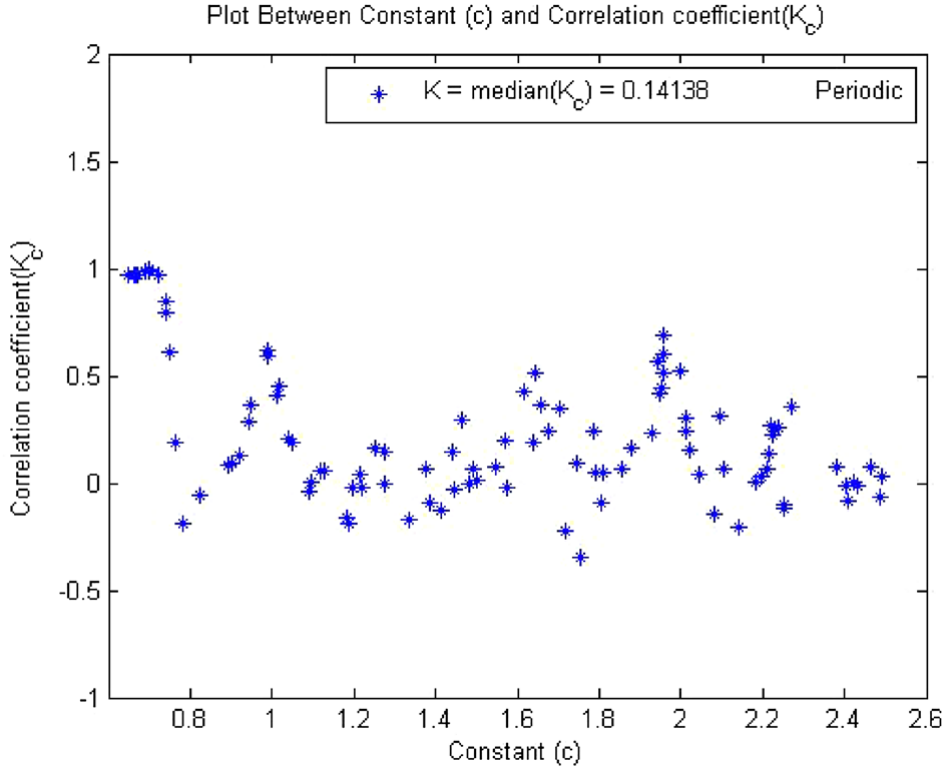
Plot of c vs $K_c$ have K = 0.141 so the dynamics ids periodic.

**Figure 12:**
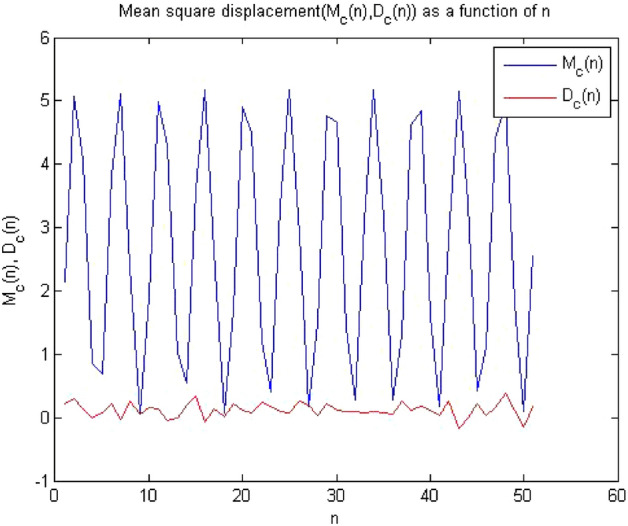
MSD vs time plot there is no asymptotic growth, so the dynamics is regular.

Figures 10, 11 and 12 shown the results of 0-1 test when time series was analyzed at $R_L={7.5}\,{\Omega }$ The forward converter had period-2 at this value of resistor. The outputs of the 0-1 test proved that the system had periodicity in its behavior as the value of indicator K approaches to zero i.e., K = 0.141, p and q graph was also bounded and there was no asymptotic growth in mean square displacement plot.

The plot in Figs. 10, 11 and 12 indicated that converter had regular dynamics as all the results showed periodicity according to the 0-1 test.

#### 0-1 test results for forward converter at period-4

**Figure 13:**
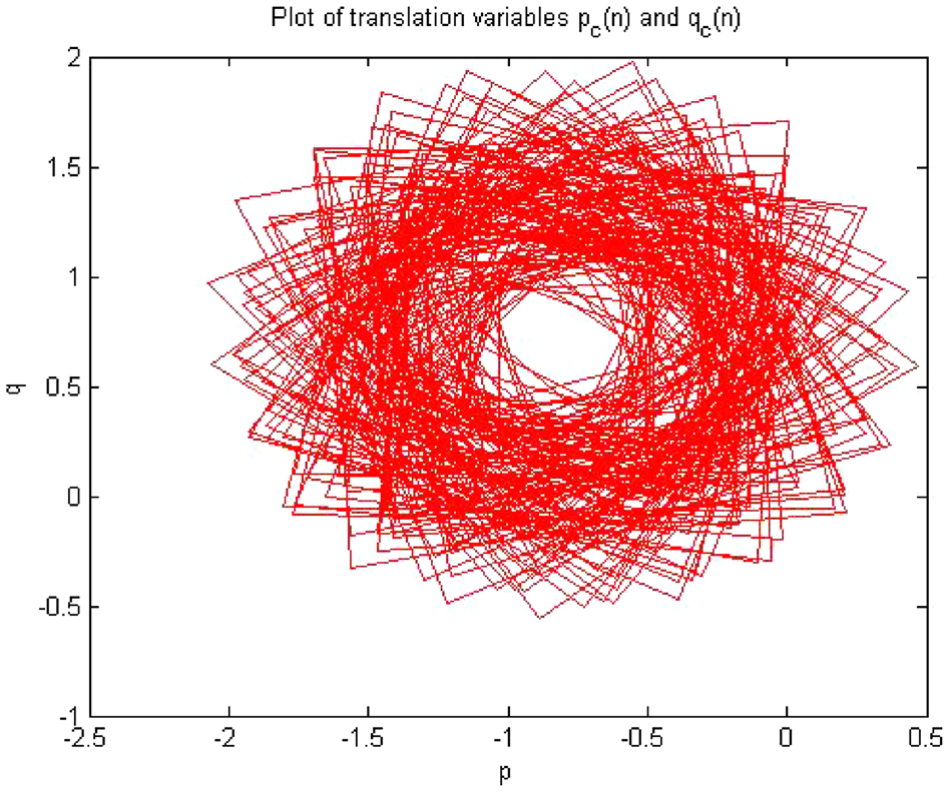
Plot of p vs q shows periodic behavior as it has bounded shape.

**Figure 14:**
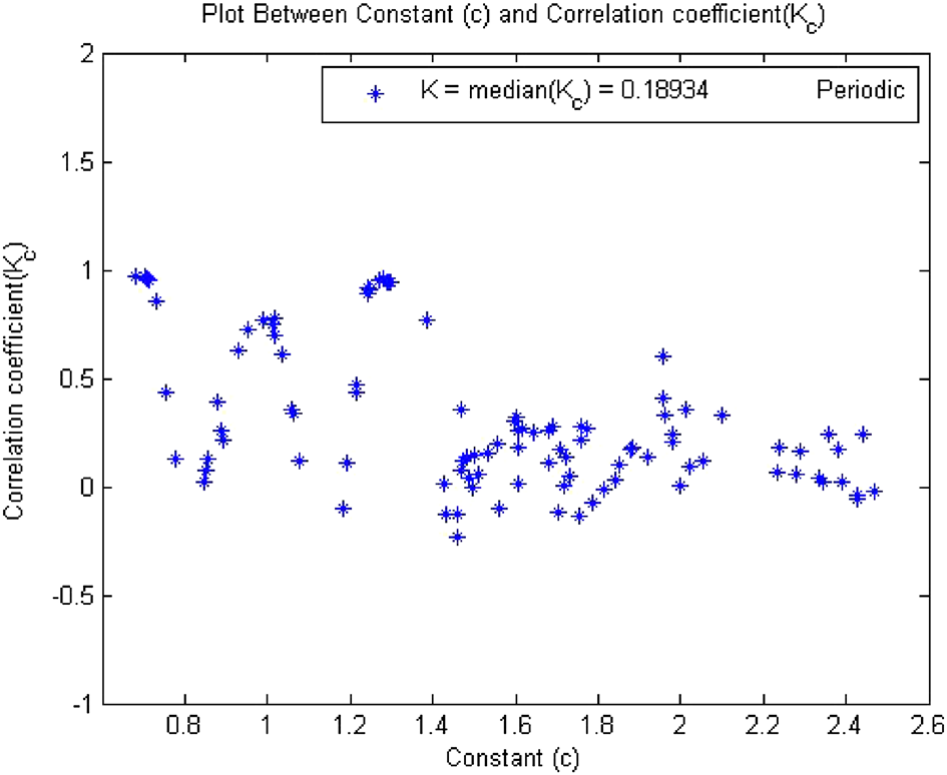
Plot of c vs $K_c$ and shows periodic behavior as K = 0.189.

**Figure 15:**
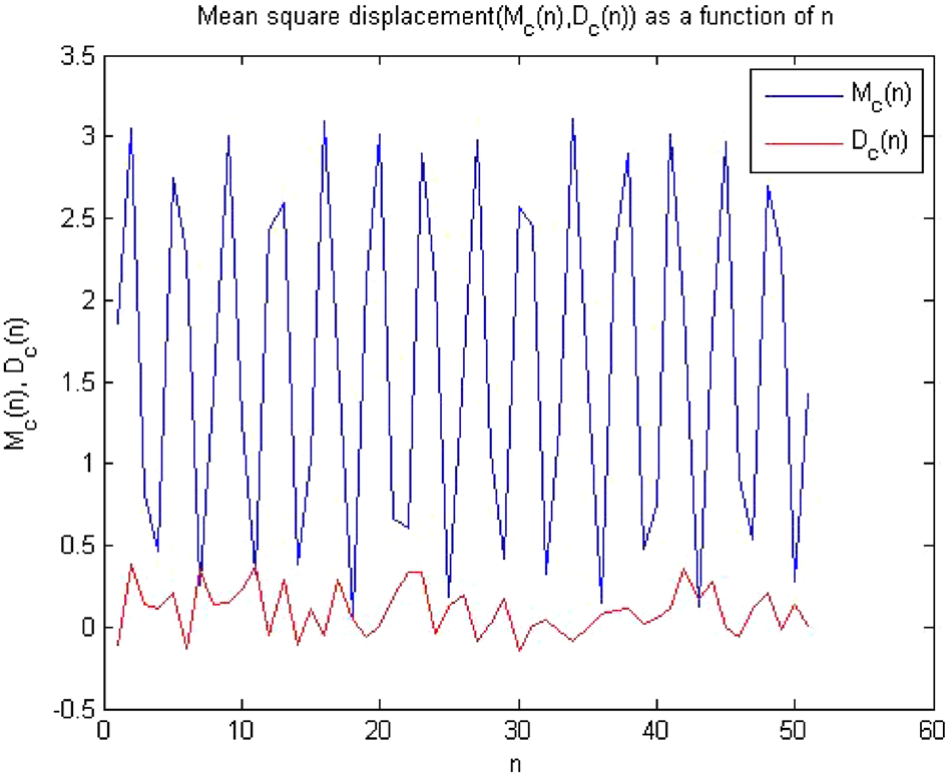
Plot of MSD vs time have periodicity as there is no asymptotic growth.

The converter showed period-4 at $R_L={9}\,{\Omega }$ , when times series at this value of load resistance were analyzed through the algorithm of 0-1 test. The results obtained has clearly indicates that the time series was periodic. Figures 13, 14 and 15 shows that the dynamics of the converter was regular and it had indicator K = 0.189, p and q graph were almost bounded and there was no asymptotic growth in mean square displacement plot. The behavior of the forward converter was regular which is evident from Figs. 13, 14 and 15. as all the plots were matched with the conditions for periodicity discussed in the methodology of 0-1 test in “Methodology”.

#### 0-1 Test results for forward converter at chaos

**Figure 16:**
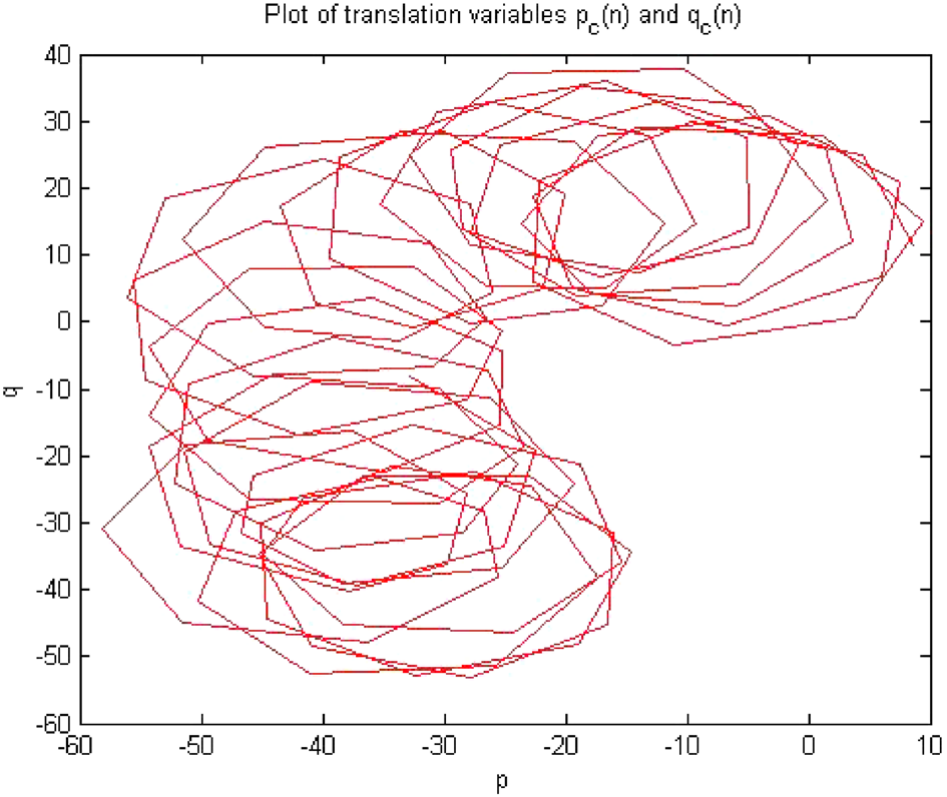
Plot of p vs q chaotic behavior it shows Brownian motion.

**Figure 17:**
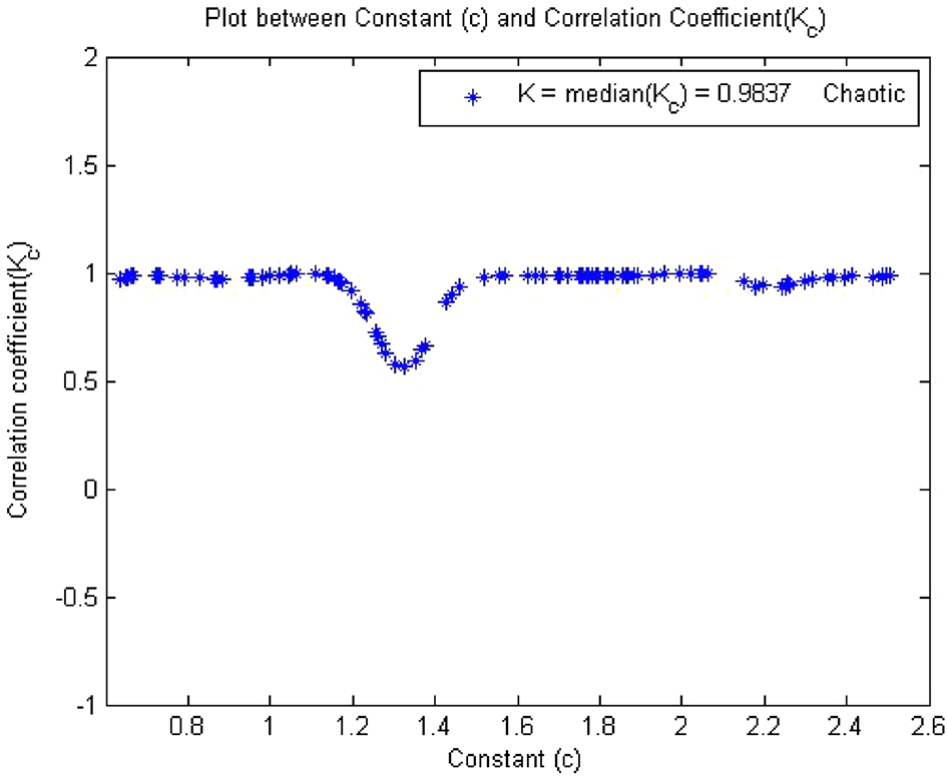
K = 0.0.983 in c vs $K_c$ plot which means dynamics is Chaotic.

**Figure 18:**
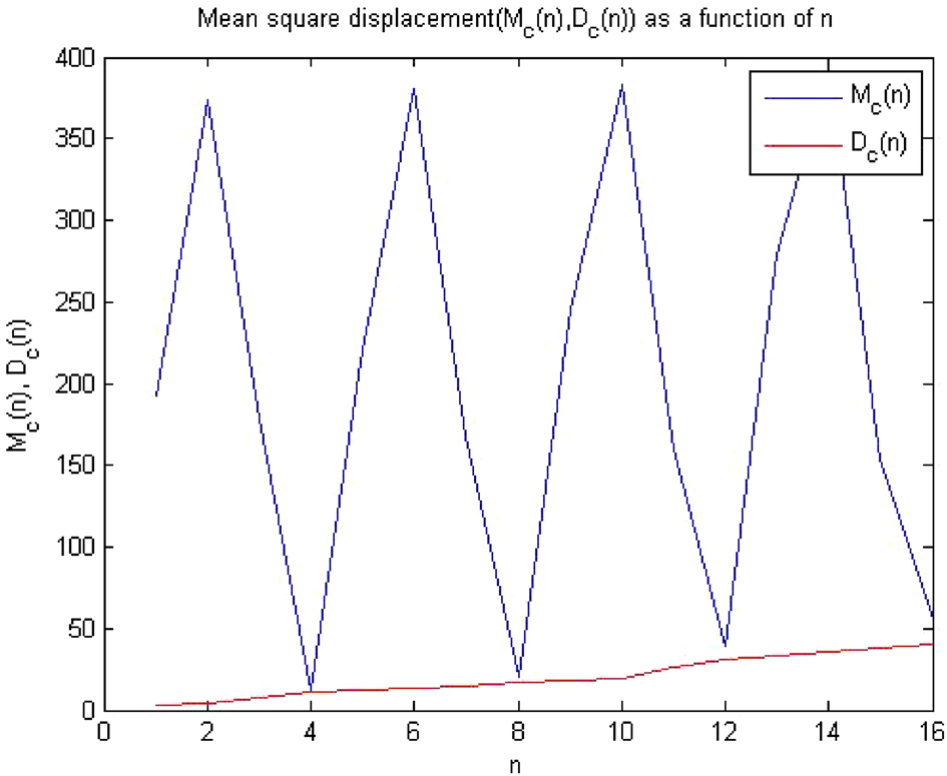
Plot of MSD corresponding to chaotic dynamics.

Times series data were obtained after the simulation circuit of forward converter at $R_L={11}\,{\Omega }$ in MultiSim, when 0-1 test algorithm was applied on this time series data, it gave the value of indicator K = 0.983, which was approximately equal to 1 shown in Fig. 16. The plot between p and q had shown Brownian motion type shape in Fig. 17 and the mean square displacement plot had also displayed asymptotic linear growth shown in Fig. 18. Hence, with the help of above results, the 0-1 test confirms that the time series was Chaotic. Therefore, forward converter had shown chaotic behavior when its load resistor was equal to $R_L={11}\,{\Omega }$. After observing the plots at at $R_L={11}\,{\Omega }$ the dynamics of the forward converter was random which is evident from Figs. 16, 17 and 18. The 0-1 test indicated system was chaotic when K was approximately 1 and p vs q plot had Brownian motion. These both conditions of 0-1 test were supported by the plots that means system was chaotic.

### Time series plot and phase portraits of forward converter

**Figure 19:**
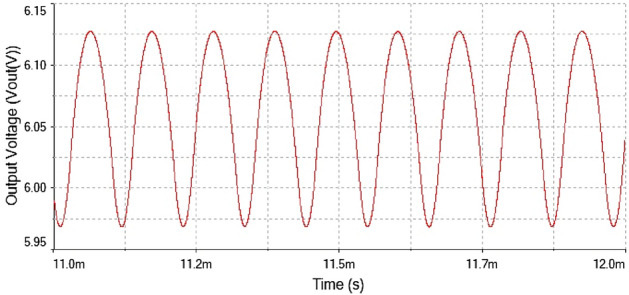
Output voltage waveform for forward converter at $R_L={4}\,{\Omega }$.

After the implementation of 0-1 test for chaos on the time series data now verified the results of 0-1 test through time series plot and phase portrait. The plots were given in Fig. 19. At $R_L={4}\,{\Omega }$ The time series plot showed regular dynamics having period-1 which is observed from the figure.

**Figure 20:**
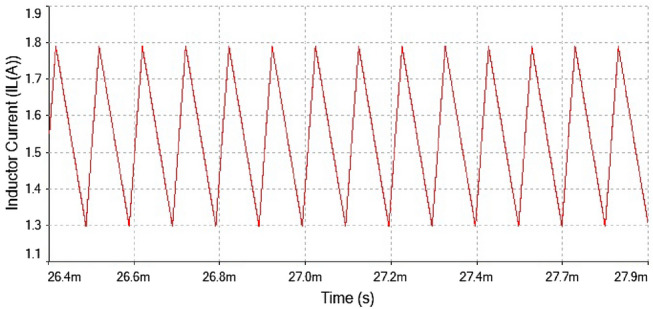
Inductor current waveform for forward converter at $R_L={4}\,{\Omega }$.

The inductor current waveform showed period-1 behavior at $R_L={4}\,{\Omega }$ is shown in Fig. 20 as the waveform is repeated after every single peak. This means forward converter had periodicity at this value of load resistance

**Figure 21:**
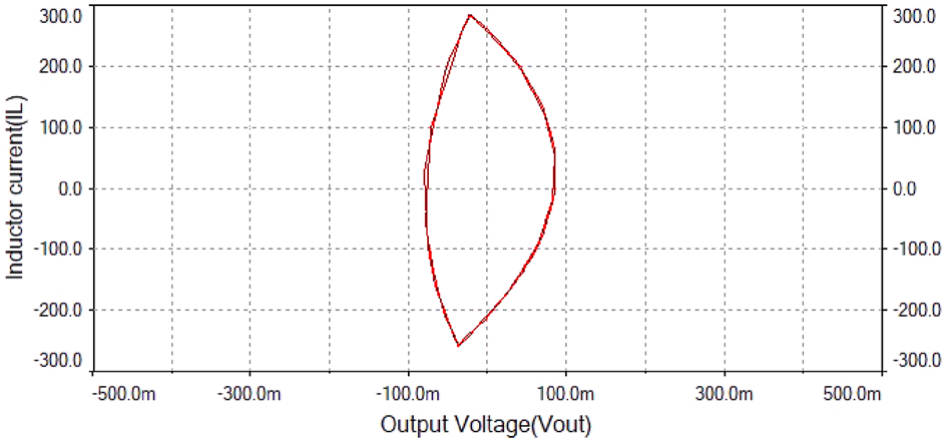
Phase portrait between $I_L$ vs $\mathrm {V}_\mathrm {out}$ at $R_L={4}\,{\Omega }$: period-1 attractor .

The phase portrait plot in Fig. 21 had single loop and it had period-1 attractor. Therefore at $R_L={4}\,{\Omega }$ the dynamics of the forward converter was regular.

Hence, it is evident from the above times series plot in Figs. 19 and 20 and phase portrait plot in Fig. 21 the forward converter showed periodic behavior having period-1 at $R_L={4}\,{\Omega }$. These results obtained from the time series and phase portrait plot were same as the results obtained from 0-1 test which was previously discussed. Hence, the converter showed periodic dynamics at this value of load resistance.

**Figure 22:**
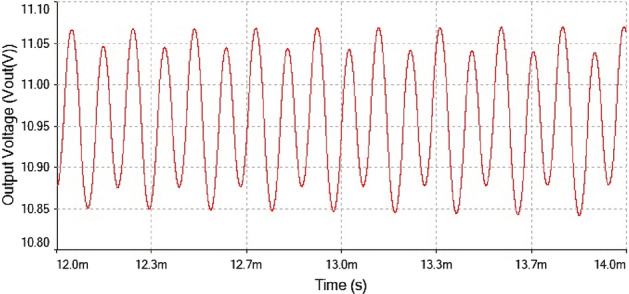
Output voltage waveform for forward converter at $R_L ={7.5}\,{\Omega }$.

In Fig. 22, the output voltage waveform is repeated after every second peak that is mean it had period-2 and when the load resistance was increased from $R_L={4}\,{\Omega }$ to $R_L={7.5}\,{\Omega }$, the converter behavior changed from period-1 to period-2.

**Figure 23:**
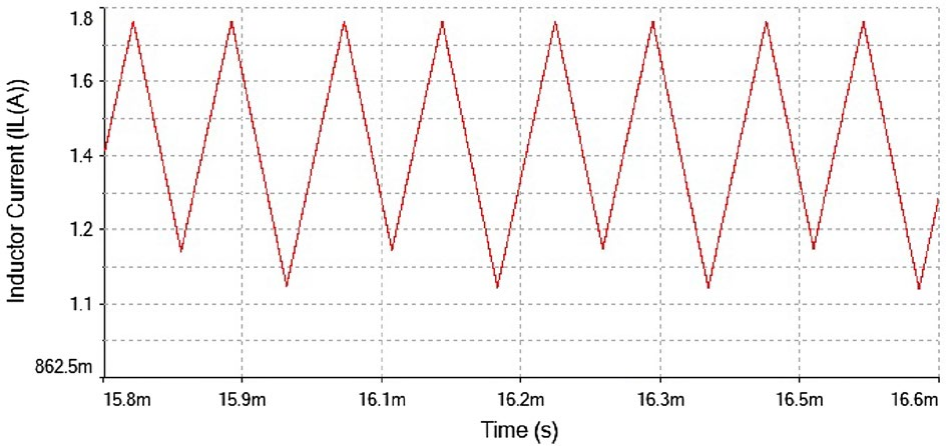
Inductor current waveform for forward converter at $R_L={7.5}\,{\Omega }$.

**Figure 24:**
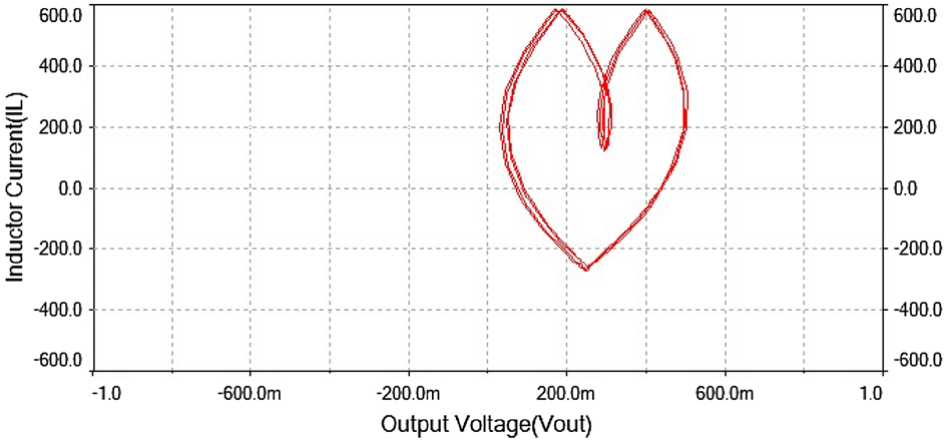
Phase portrait between $I_L$ vs $\mathrm {V}_\mathrm {out}$ at $R_L={7.5}\,{\Omega }$: period-2 attractor .

The inductor current time series waveform shown in Fig. 23. the waveform showed period-2 behavior because it repeated itself after every two spikes. This also confirmed that at $R_L={7.5}\,{\Omega }$ had regular behavior. The phase portrait plot between inductor current and output voltage also indicated the converter had period-2 at $R_L={7.5}\,{\Omega }$ which is shown in Fig. 24.

In Figs. 22 and 23 for $R_L={7.5}\,{\Omega }$ the inductor current $(I_L)$ and output voltage ($\mathrm {V}_\mathrm {out}$) were shown. It had been observed from the time series waveform that it has period-2. The phase portrait plot had also conformed period doubling phenomenon was shown in Fig. 24.

Hence, from the results obtained from phase portrait plot and time series at $R_L={7.5}\,{\Omega }$ confirmed the results obtained from 0-1 test at period-2, which showed that the forward converter had periodic behavior at this value of load resistance.

**Figure 25:**
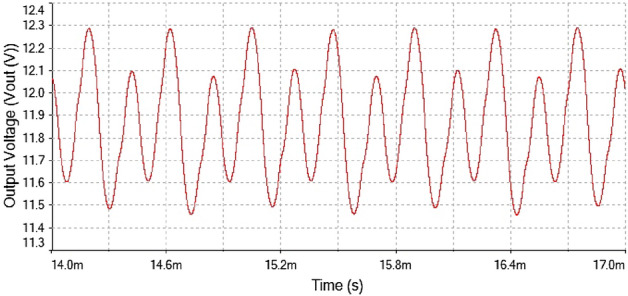
Output voltage waveform for forward converter at at$R_L={9}\,{\Omega }$.

**Figure 26:**
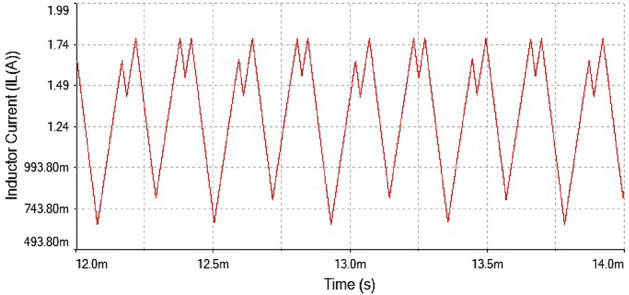
Inductor current waveform for forward converter at $R_L={9}\,{\Omega }$.

The output voltage time series waveform displayed period-4 by visual inspection of Fig. 25 and converter was still having some periodicity in its dynamics at $R_L={9}\,{\Omega }$. Similarly, the forward converter showed period-4 which was visualized from its inductor current waveform at $R_L={9}\,{\Omega }$ in Fig. 26.

**Figure 27:**
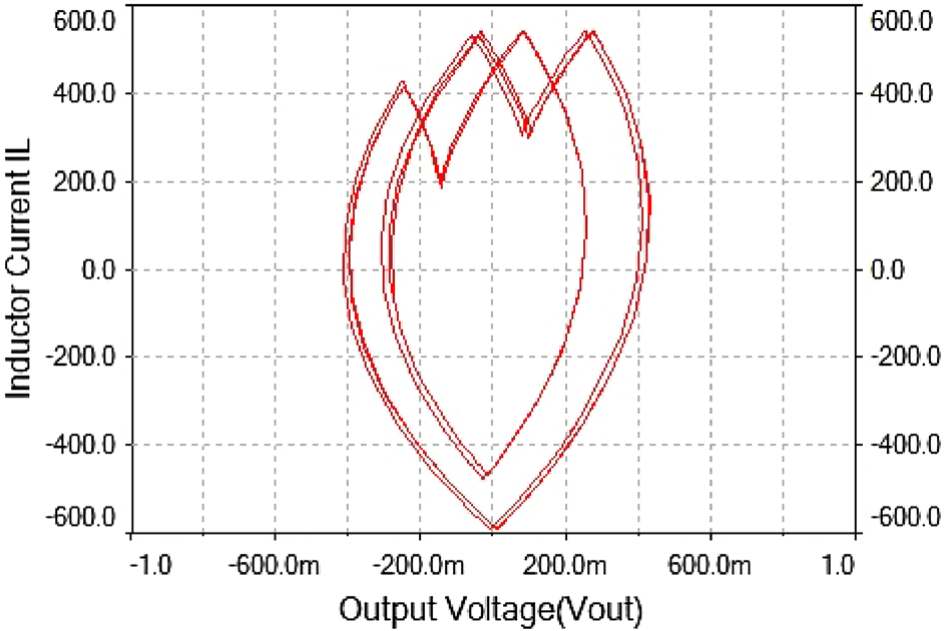
Phase portrait between $I_L$ vs $\mathrm {V}_\mathrm {out}$ at $R_L={9}\,{\Omega }$: period-4 attractor .

**Figure 28:**
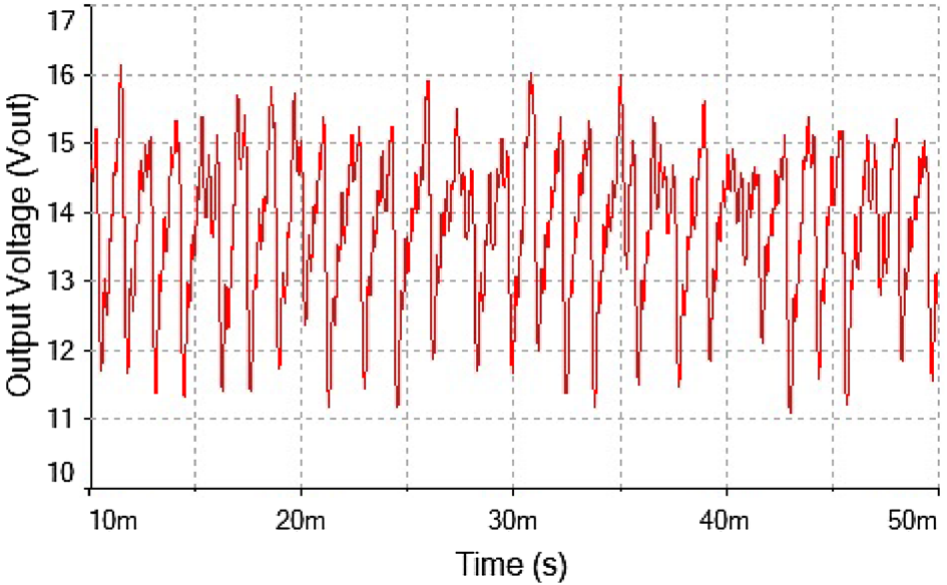
Output voltage waveform for forward converter at at$R_L={11}\,{\Omega }$.

The phase portrait plot between inductor current and output voltage shown in Fig. 27 had fourth periodic attractor at $R_L={9}\,{\Omega }$. The forward converter has period-4 at $R_L={9}\,{\Omega }$ which is clearly visible from the time series waveform in Figs. 25 and 26. The phase portrait plot also has four loop which indicates that it had period-4 shown in Fig. 27. Hence, from the plot shown in the above figure matched with 0-1 test results in the previous section. The waveform shown in Fig. 28 is the time series plot of output voltage at $R_L={11}\,{\Omega }$. The waveform clearly indicate there is no repetition. Hence, forward converter showed random behavior at this value of load resistance. The inductor current waveform shown chaotic phenomenon as there is on regular repetition of the time series waveform displayed in Fig. 27. Thus, the froward converter had random behavior at $R_L={11}\,{\Omega }$.

**Figure 29:**
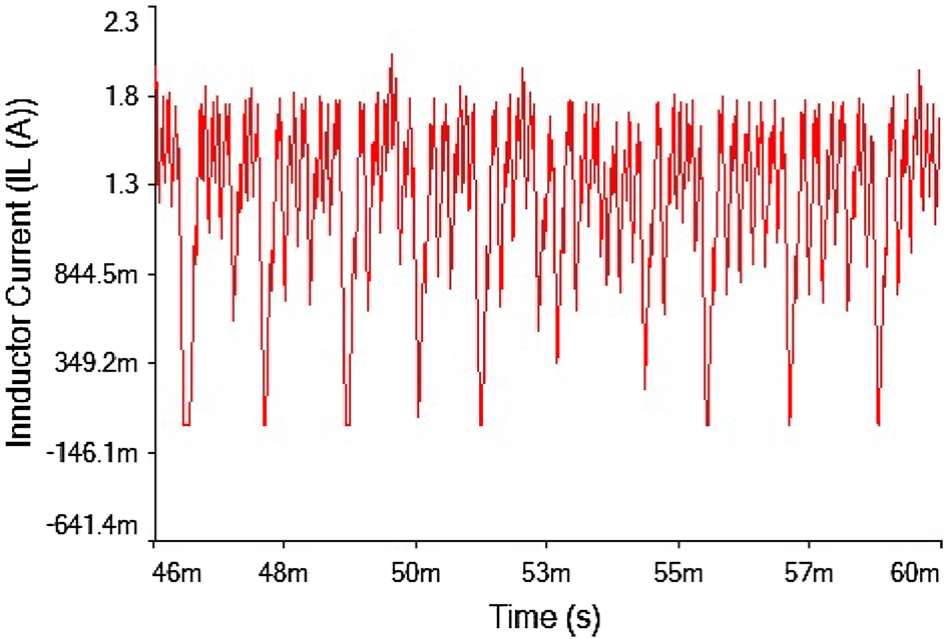
Inductor current waveform for forward converter at $R_L={11}\,{\Omega }$.

**Figure 30:**
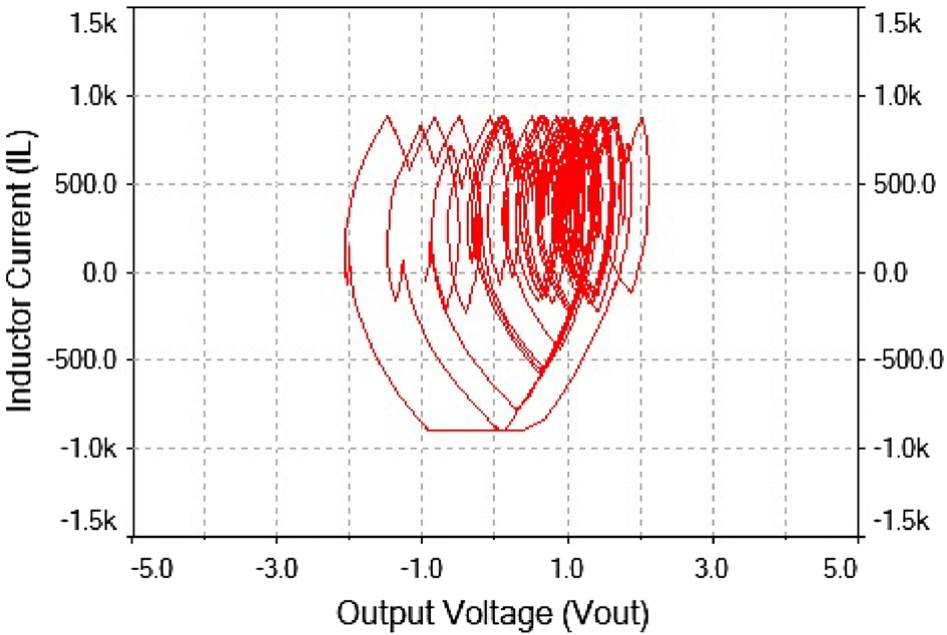
Phase portrait between $I_L$ vs $\mathrm {V}_\mathrm {out}$ at $R_L={11}\,{\Omega }$: chaotic attractor.

The phase portrait plot between inductor current and output voltage has strange attractor as seen from Fig. 30. Hence, forward converter had chaotic behavior at $R_L={11}\,{\Omega }$ The time series waveform were obtained at $R_L={11}\,{\Omega }$ which is showed in Figs. 28 and 29, that these waveform were aperiodic. The phase portrait also has showed a strange attractor in Fig. 30. thus, by increasing the load resistance the converter route to chaos. Hence, results obtained from the 0-1 test proved right by these phase portraits and time series plots in the figures discussed above.

### Effects of number of data points on 0-1 test results

**Figure 31:**
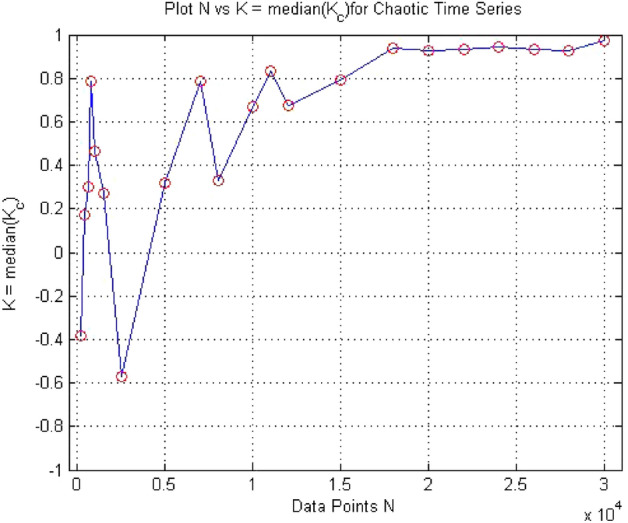
Plot of K as a function of N for the forward converter at $R_L={11}\,{\Omega }$.

0-1 test is required a large amount data points to avoid false results. The effect of number of data points had been discussed in “Methodology”. Figure 31 shows how the value of K depends on the amount of data points used. By observing the plot, it had been cleared that at lower data points the test gives value of k close to zero, whereas time series was chaotic in nature, but gradually increase in data points the test results start approaches to 1. Therefore, the test depends upon number of data point and abundance of data points required to avoid spurious results. In this study, 50,000 data points had been used for forward converter to avoid false results.

### Oversampling of continuous time series data

**Figure 32:**
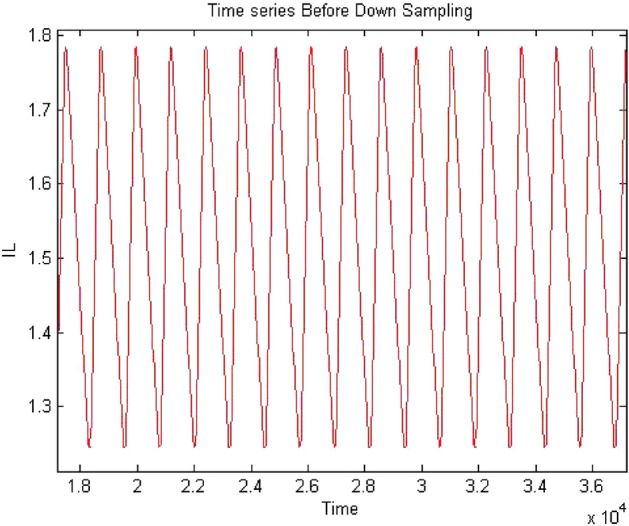
Time series of forward converter at period-1 before down sampling.

**Figure 33:**
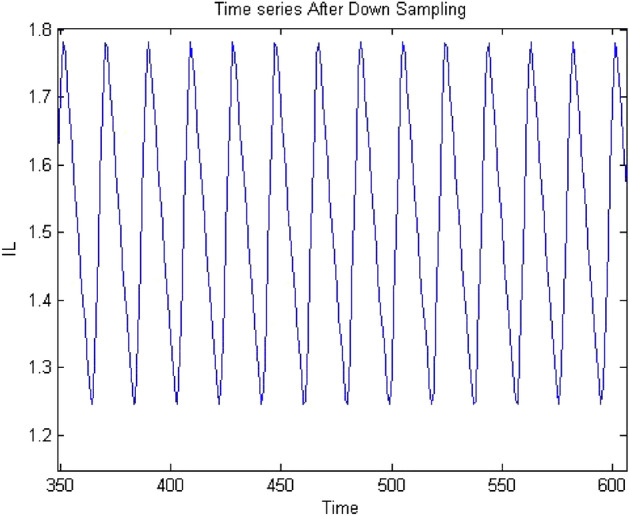
Time series of forward converter at period-1 after down sampling.

**Figure 34:**
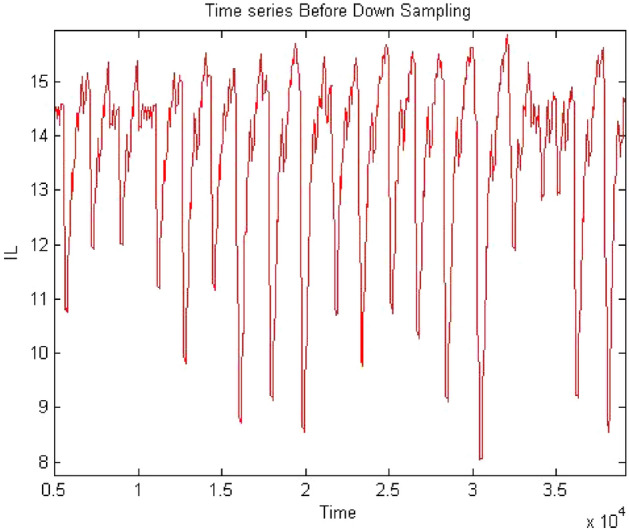
Time series of forward converter at chaotic before down sampling.

**Figure 35:**
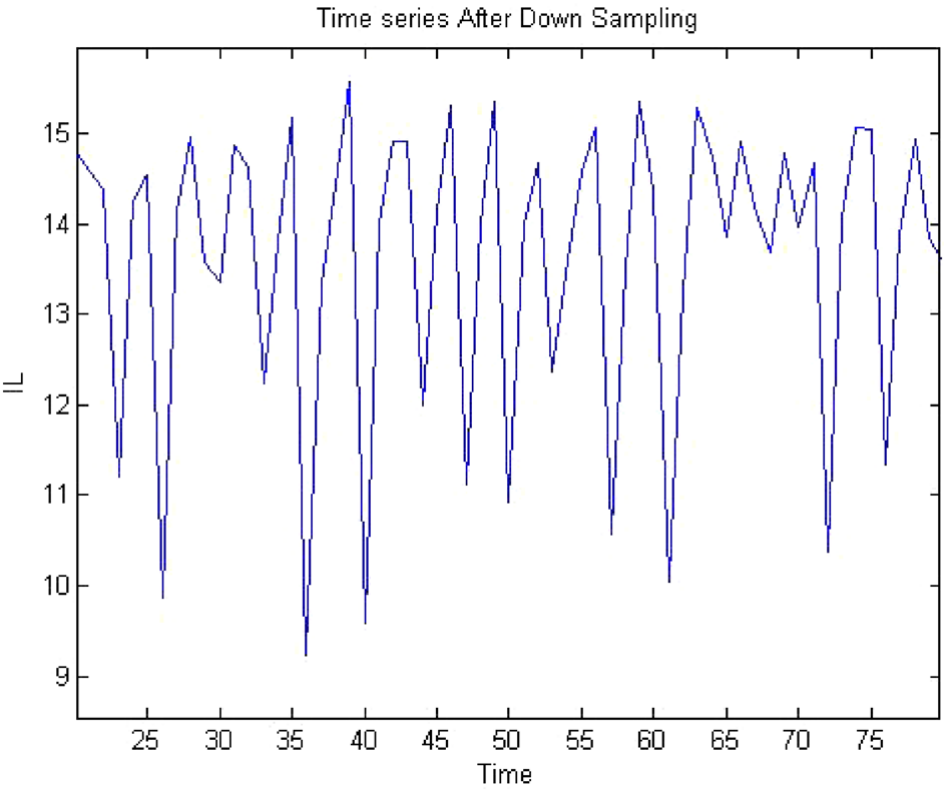
Time series of forward converter at chaotic after down sampling.

Forward converter is continuous systems. The time series obtained from continuous system may raise oversampling issue when 0-1 test implemented on it. Therefore, it may require to down sample the time series data to apply the test. The sampling needs to be done in such a way that it does not affect the dynamics of the system and require chose the sampling time appropriately. The method of chose sampling instance was discussed in “Methodology” detail. In this study the time series were down sampled and after down sampling when check the behavior of time series it retained on its original dynamics. To address oversampling issue in continuous dynamical system had been discussed in “Methodology”. In this section, discussed the effect of sampling on time series. The times series data had obtained at $R_L={4}\,{\Omega }$ having period-1 for forward converter before down sampling as shown in Fig. 32 and waveform had remained periodic even after down sampled as shown in Fig. 33. Similarly, in Fig. 34 the forward converter waveform at $R_L={11}\,{\Omega }$ was shown before down sampling, which was chaotic in behavior and when this time series had been down sampled the waveform has continued its chaotic behavior as shown in Fig. 35. hence, it was concluded that sampling had no effect on the dynamics of the system.

## Conclusion

The power electronic device has vastly applied in power distribution system, energy storage system and renewable energy resources.This research is based on the analysis of the non-linear dynamics of forward converter via 0-1 test. 0-1 test has been implementation on forward converter to study its inherent non-linear dynamics. The simulation results have revealed that the forward converter undergoes period-1, period-2, period-4, and chaos with the increase in load resistance.

**Table 2:** Solution of dynamics of the forward converter.

Solution	Periodic	Period doubling	Quasi periodic	Chaotic
Time series	Repeat after single peak	Repeat after two peaks	Repeat after four peaks	Noise
Phase portrait	Single Loop	Double loop	Torus	Distinct shapes
Bifurcation diagram	Single branch	Double the branches	Quadruple the branches	Multiples branches
0-1 test	K $\approx$ 0	K $\approx$ 0	K $\approx$ 0	$K \approx$ 1

The 0-1 test for chaos is used to analysis the nonlinear continuous and discrete deterministic dynamical systems. This test distinguishes between regular and chaotic dynamics of the system and it gives the value of median K approximately equal to 1 for the chaotic system and closed to zero for the regular system. It only requires the time series data. The important aspect this test is that it does not require phase portrait reconstruction and it is independent of nature of the vector field, which has observed during the implementation of test on forward converter. The test results have also been supported via bifurcation diagram, Poincare map, time series plot and phase portrait plot. The dynamics of the system were analyzed through different tool for detecting chaos which include times series plot, phase portrait plot, bifurcation diagram, Poincare map and 0-1 test and their results were compared which is given in Table 2.

In future, this study will help the researcher to design an optimal controller for the forward converter. The efficient controller is required to enhance the performance of the forward converter while it is utilizing in the modern electrical systems. The nonlinearity emerges in the forward converter because of the varying load must be suppress for optimal operation (Supplementary Information).

## Supplementary Information

Supplementary Information.

## Data Availability

The data sets generated during and/or analyzed during the current study are available from the corresponding author on reasonable request.
