# Supplementary material for: Application of 0-1 test for chaos on forward converter to study the nonlinear dynamics
Source: Sci Rep. 2022 Sep 20;12:15696. doi: 10.1038/s41598-022-19667-7 (PMC9489791; doi:10.1038/s41598-022-19667-7)
Supplement: Supplementary file 1 — Supplementary Information. [file 41598_2022_19667_MOESM1_ESM.pdf]

APPENDIX

0-1 test Equations

Formulation of Translation Variable p_c and q_c

The time series vector ϕ(j), j = 1, …, N, which is extracted during the experiment process are used by 0-1 and with help of ϕ(j) define p_c and q_c as translation variable and define as

$$p_c = \sum_{j=1}^{n} \phi(j)\cos(jc) \quad (1)$$

$$q_c = \sum_{j=1}^{n} \phi(j)\sin(jc) \quad (2)$$

for n = 1, …, N, and c ϵ (0, π) it is randomly chosen real number [1,2]. Then draw the plot between p_c and q_c show the system show the periodicity or chaotic dynamics. If the plot is bounded, then the system has regular dynamics otherwise if there is Brownian motion then its dynamics is chaotic [1-4].

Selection of c:

The value of c at some point created resonance like phenomenon that produce false result. In order to neutralize this occurrence Gottwald and Melbourne recommended to choose the value of c in the limited interval (π/5, 4 π/5) [2,3].

Calculation of Mean Square Displacement (MSD)

To compute the value of K, first determine the mean square displacement (MSD).

$$M_c(n) = \lim_{N\to\infty} \frac{1}{N} \sum_{j=1}^{N} ([p_c((j+n) - p_c(j)]^2 + [q_c(j+n) - q_c(j)]^2) \quad (3)$$

In this n ≪ N required. The equation (4) are used to calculate mean square displacement. The limit is assured for calculating $M_c(n)$ only for n ≤ n_{cut} where n_{cut}≪ N and n_{cut} = N/10 to get good results. The $p_c(n)$ and $q_c(n)$ have oscillating component which was removed by using linear transformation of the $M_c(n)$ which is explained in [2,5]as given

$$Mc(n) = V(c)\,n + Vosc(c,n) + e(c,n) \quad (4)$$

where $\frac{e(c,n)}{n} \rightarrow 0$

as n → ∞ uniformly in c ∈ (0, π)

$$V_{osc}(c,n) = (E\emptyset)^2 \frac{1-\cos(nc)}{1-\cos(c)} \quad (5)$$

Subtract V_{osc} (c, n) we get Modified mean square displacement [6].

$$\mathrm{D_c(n) = M_c(n) - V_{osc}(c.n)} \quad (6)$$

which exhibits the same asymptotic growth of $\mathrm{M_c(n)}$ but with better convergence [2].

Method to Calculate Kc

The analysis of time series via 0-1 test whether it is chaotic or periodic through regression or correlation method [2]. The numerical result show either 0 or as a value of K, if K≈1 then chaotic or K≈0 periodic [6,7]. There are two methods to calculate Kc.

i. Regression Method

$$K_r = \lim_{n\to\infty} \frac{logMc(n)}{\log n} \quad (7)$$

$$\mathrm{D^{\sim}{}_c(n) = D_c(n) + a \min_{1\le n\le N_o} |D_c(n)|} \quad (8)$$

$\mathrm{D^{\sim}{}_c(n)}$= Dc(n) +a min 1≤n≤N0 |Dc(n)| (9)

where $a > 1$ to get the asymptotic growth rate

$$K_r{}^* = \lim_{n\to\infty} \frac{log\mathrm{D^{\sim}{}_c(n)}}{\log n} \quad (10)$$

ii. Correlation Method

On contrary to this if we use correlation method to calculate Kc then first, we create two vectors $\xi = \{1, 2, \ldots, n_{cut}\}$ [2,3].

$M = \{Mc (1), Mc (2), \ldots, Mc(n_{cut})\}$.

or

$D = \{Dc (1), Dc (2), \ldots, Dc(n_{cut})\}$.

The two-vector x and y given above having length r and their covariance as:

$$\mathrm{cov(x,y)} = \frac{1}{q}\sum_{j=1}^{q}(\mathrm{x(j)} - \bar{\mathrm{x}})(\mathrm{y(j)} - \bar{\mathrm{y}}) \quad (11)$$

$$where\ \bar{x} = \frac{1}{q}\sum_{j=1}^{q} x(j)$$

To find the correlation constant K_c

$$K_c = \mathrm{corr}(\xi.\Delta) = \frac{\mathrm{cov(\xi.M)}}{\sqrt{\mathrm{var(\xi)var(M)}}}\epsilon(-1,1) \quad (12)$$

or

$$K_c{}^* = \mathrm{corr}(\xi.\Delta) = \frac{\mathrm{cov}(\xi.\mathrm{D})}{\sqrt{\mathrm{var}(\xi)\mathrm{var}(\mathrm{D})}} \epsilon(-1,1) \quad (13)$$

References

1 Sun, K.-H., Liu, X. & Zhu, C.-X. The 0-1 test algorithm for chaos and its applications. *Chinese Physics B* **19**, 110510 (2010).

2 Gottwald, G. A. & Melbourne, I. On the implementation of the 0–1 test for chaos. *SIAM Journal on Applied Dynamical Systems* **8**, 129-145 (2009).

3 Gottwald, G. A. & Melbourne, I. The 0-1 test for chaos: A review. *Chaos detection predictability*, 221-247 (2016).

4 Melosik, M. & Marszalek, W. On the 0/1 test for chaos in continuous systems. *Bulletin of the Polish Academy of Sciences: Technical Sciences* (2016).

5 Bernardini, D. & Litak, G. An overview of 0–1 test for chaos. *Journal of the Brazilian Society of Mechanical Sciences Engineering* **38**, 1433-1450 (2016).

6 Gopal, R., Venkatesan, A. & Lakshmanan, M. Applicability of 0-1 test for strange nonchaotic attractors. *Chaos: An Interdisciplinary Journal of Nonlinear Science* **23**, 023123 (2013).

7 Gottwald, G. A. & Melbourne, I. Testing for chaos in deterministic systems with noise. *Physica D: Nonlinear Phenomena* **212**, 100-110 (2005).
